# Influence of the combination and phase variation status of the haemoglobin receptors HmbR and HpuAB on meningococcal virulence

**DOI:** 10.1099/mic.0.046946-0

**Published:** 2011-05

**Authors:** Isfahan Tauseef, Odile B. Harrison, Karl G. Wooldridge, Ian M. Feavers, Keith R. Neal, Stephen J. Gray, Paula Kriz, David P. J. Turner, Dlawer A. A. Ala’Aldeen, Martin C. J. Maiden, Christopher D. Bayliss

**Affiliations:** 1 Department of Genetics, University of Leicester, Leicester LE1 7RH, UK; 2 The Department of Zoology, University of Oxford, South Parks Road, Oxford OX1 3SY, UK; 3 Molecular Bacteriology and Immunology Group, University of Nottingham, Nottingham, UK; 4 National Institute for Biological Standards and Control, Blanche Lane, South Mimms, Hertfordshire EN6 3QG, UK; 5 School of Community Health Sciences, University of Nottingham, Nottingham, UK; 6 Health Protection Agency, Meningococcal Reference Unit, Manchester Royal Infirmary, Manchester M13 9WL, UK; 7 National Reference Laboratory for Meningococcal Infections, National Institute of Public Health, Prague, Czech Republic

## Abstract

*Neisseria meningitidis* can utilize haem, haemoglobin and haemoglobin–haptoglobin complexes as sources of iron via two TonB-dependent phase variable haemoglobin receptors, HmbR and HpuAB. HmbR is over-represented in disease isolates, suggesting a link between haemoglobin acquisition and meningococcal disease. This study compared the distribution of HpuAB and phase variation (PV) status of both receptors in disease and carriage isolates. Meningococcal disease (*n* = 214) and carriage (*n* = 305) isolates representative of multiple clonal complexes (CCs) were investigated for the distribution, polyG tract lengths and ON/OFF status of both haemoglobin receptors, and for the deletion mechanism for HpuAB. Strains with both receptors or only *hmbR* were present at similar frequencies among meningococcal disease isolates as compared with carriage isolates. However, >90 % of isolates from the three CCs CC5, CC8 and CC11 with the highest disease to carriage ratios contained both receptors. Strains with an *hpuAB*-only phenotype were under-represented among disease isolates, suggesting selection against this receptor during systemic disease, possibly due to the receptor having a high level of immunogenicity or being inefficient in acquisition of iron during systemic spread. Absence of *hpuAB* resulted from either complete deletion or replacement by an insertion element. In an examination of PV status, one or both receptors were found in an ON state in 91 % of disease and 71 % of carriage isolates. We suggest that expression of a haemoglobin receptor, either HmbR or HpuAB, is of major importance for systemic spread of meningococci, and that the presence of both receptors contributes to virulence in some strains.

## Introduction

*Neisseria meningitidis* (the meningococcus) is an obligate commensal of humans, residing in the nasopharynx of 10–30 % of individuals. Meningococci can invade host tissues and disseminate in blood, causing meningitis and septicaemia, which are leading causes of mortality among children and young adults worldwide (Pollard, 2004; Stephens, 2007). Studies in animal models have demonstrated that the virulence of pathogenic meningococci increases significantly when bacteria are injected in combination with iron complexes (Perkins-Balding *et al.*, 2004). In humans, iron is usually sequestered in complexes with iron-binding proteins such as transferrin, lactoferrin and haemoglobin (Otto *et al.*, 1992). Meningococci encode several surface receptors to strip and acquire iron or haem from these iron-binding proteins, including two TonB-dependent haem acquisition systems, HpuAB and HmbR (Perkins-Balding *et al.*, 2004). HpuAB is a bipartite complex consisting of a lipoprotein, HpuA (37 kDa), and a transmembrane protein, HpuB (85 kDa), both of which are required to bind haptoglobin–haemoglobin complexes and free haemoglobin (Lewis & Dyer, 1995; Rohde & Dyer, 2004). HmbR is an 89 kDa transmembrane protein that specifically binds haemoglobin (Perkins-Balding *et al.*, 2003). This redundancy in haemoglobin binding receptors may enable meningococci to acquire haem from a variety of sources and niches or may facilitate immune evasion.

The distribution of HpuAB and HmbR varies among meningococcal strains, with some containing both systems and others only one (Lewis *et al.*, 1999; Richardson & Stojiljkovic, 1999). Recent studies have detected a significantly higher frequency of *hmbR* in disease as compared with carriage isolates, suggesting that this receptor contributes to meningococcal pathogenesis (Harrison *et al.*, 2009). The expression of these systems is phase variable due to stretches of polyG nucleotides present in the ORFs of *hpuA* and *hmbR* (Lewis *et al.*, 1999; Richardson & Stojiljkovic, 1999). Mutations in these tracts arising by slipped-strand mispairing during DNA replication lead to ‘ON’ and ‘OFF’ switches in the expression of these receptors. Differential expression due to changes in tract length helps bacteria to adapt to fluctuations in their microenvironments (Moxon *et al.*, 2006), and may also contribute to escape from the adaptive immune responses within the host (Bayliss *et al.*, 2008). Immune evasion may also be facilitated by significant levels of antigenic diversity in HmbR (Evans *et al.*, 2010). Understanding the contributions of these haemoglobin receptors to meningococcal disease and the impact of immune responses on these surface proteins is hampered by a lack of detailed studies on the distribution, antigenic variation and phase variation (PV) status of the two receptors in epidemiological samples from disease and carriage.

## Methods

### 

#### Bacterial isolates, culture conditions and DNA extraction.

Isolates from four collections were analysed. The first group comprised 107 isolates assembled globally between 1937 and 1996 (Maiden *et al.*, 1998). The second group contained 88 carriage isolates collected in 2008 from first year students at Nottingham University (Bidmos *et al.*, 2011). The third collection included 153 isolates assembled in the Czech Republic in 1993 (Jolley *et al.*, 2000). The fourth group contained 77 disease and 82 carriage isolates collected in England and Wales between 1999 and 2000 (from the Health Protection Agency Meningococcal Reference Unit, Manchester, UK, and the UK Carriage Study; Maiden *et al.*, 2002). Twelve additional sequence type (ST)-41/44 isolates from the UK were also analysed (provided by I. M. Feavers). Bacteria were grown overnight, followed by either preparation of bacterial lysates or chromosomal DNA extraction using a DNeasy Blood and Tissue kit (Qiagen). Isolates were classified as disease or carriage depending on whether they were isolated from patients or carriers, respectively.

#### PCR amplification and sequence analysis.

The presence of *hmbR* was detected with primers hmbR-RF3 (5′-TGCCAACCTCTTTTACGAATGG-3′) and hmbR-RF4 (5′-GCTACTGAACACGTCGTTCC-3′), and that of *hpuAB* with primers hpuAC (5′-ATGCGATGAAATACAAAGCCC-3′) and hpuA350Rev (5′-GGATGAAAGGGCGTATTGCGC-3′) or hpuAC and P26.85 (5′-GGGAAACGCTTGGGCGATGG-3′). Isolates negative for *hpuAB* were screened with primers hpu-for1 (5′-GCAACAATGCCTTGTCATCC-3′) and hpu-rev13 (5′-TGATCGAAATGGGCGTACTC-3′), which bind on either side of the *hpuAB* locus. For analyses of *hpuAB* deletion and replacement events, hpu-for1/hpu-rev13 amplicons were sequenced with primers hpu-for1, hpu-rev13, hpuF-seq (5′-GGCAACTTTTCCACCGTCATTC-3′) and hpuF-seq2 (5′-AAACCGGCAACATCTGGAAG-3′). Complete sequences for HpuA were determined by amplification with N- and C-terminal primers, followed by sequencing of the products with both PCR primers and internal primers.

Repeat numbers for the phase-variable polyG tracts of *hmbR* and *hpuA* were determined by sequencing of the amplicons utilized for detection of these genes and enumeration of G residues in the tracts contained in this sequence data. For *hpuA*, a 10G tract is associated with an intact reading frame in isolates Z2491 and FAM18, and hence represents the ON status. Similarly, *hmbR* is in an ON state if the tract is 9G, as found in strain MC58. The alignment of sequences from test isolates with the Z2491 and MC58 sequences confirmed the ON/OFF status and showed that none of the insertion/deletion events around the repeat tracts affected the reading frame. The ON/OFF status for *hpuA* was also confirmed for 10 isolates by translating complete gene sequences.

The tract lengths and ON/OFF status of some isolates from the 2008 carriage study were determined by a combination of sequencing and sizing of PCR fragments. Isolates were assigned to clones on the basis of identical *porA*, *fetA* and MLST types, and then the repeat tracts of *hmbR* and *hpuA* were determined by sequencing for one or two isolates per clone. These genes were then amplified from all isolates of each clone with a 6-carboxyfluorescein (FAM)-labelled primer, hpuA-350Rev or hmbR-RF3, and the relevant non-labelled primer. PCR products were subjected to A-tailing by addition of a 4 µl reaction mix containing 0.4 µl PCR buffer (10×), 0.4 µl MgCl_2_ (25 mM), 0.05 µl *Taq* and 3.15 µl distilled H_2_O, followed by incubation at 72 °C for 45 min. Diluted PCR products (0.5 µl) were then mixed with 0.5 µl GeneScan 500 LIZ size standard (Applied Biosystems) and 9 µl formamide, followed by denaturation and electrophoresis on an Autosequencer (Applied Biosystems). GeneScan data were analysed using Peak Scanner v1.0 software (Applied Biosystems).

#### Phenotypic analysis of phase variants.

Meningococcal strains were grown overnight on brain heart infusion (BHI) plates containing Levinthal’s supplement. Suspensions of bacteria were prepared and spread onto Mueller–Hinton agar (MHA) plates containing 40 µM desferal and onto plain MHA plates. Disks were impregnated with 10 µl of either human haemoglobin (10 mg ml^−1^, Sigma) or transferrin (50 mg ml^−1^, Sigma) and placed onto a desferal-containing MHA plate along with a third disk without any added iron source. Plates were incubated overnight at 37 °C in 5 % CO_2_. Confluent growth was observed on the plain MHA plates but was absent on plates containing desferal and no added iron source.

#### Statistical analyses.

Statistical analyses were carried out using GraphPad Prism version 5. Odds ratios (ORs) and 95 % confidence intervals (CIs) were derived using a Chi-squared test and *P* values with a Fisher’s exact test.

## Results

### Sequence variation of HpuA

The variability in the amino acid sequences of the surface-exposed component of the HpuAB receptor, HpuA, has not been reported previously. Complete sequences of *hpuA* were generated for a serogroup B isolate (strain 8047) and isolates representing the following clonal complexes (CCs): 23, 60, 167 and 174. The nucleotide and derived amino acid sequences were aligned with those extracted from the Z2491 and FAM18 genome sequences (Supplementary Fig. S1). HpuA proteins varied in size from 326 to 342 aa due to a series of eight indels, including a large insertion of 16 aa within the N-terminal region, and exhibited significant levels of variation (76–94 % identity), with four major variable regions and 101 polymorphic sites. As nothing is known about the functional domains of this protein, it is unclear whether this variability will influence its functions or antigenicity. A similarly high level of variability was observed for TbpB, the outer membrane component of the meningococcal transferrin-binding protein, although this variability did not alter transferrin binding by this protein (Boulton *et al.*, 1998).

### Distribution of haemoglobin receptors in carriage and disease isolates

An epidemiological study of the distribution of one of the haemoglobin receptors, HmbR, has revealed a significantly higher frequency of this gene in meningococcal disease isolates compared with carriage isolates (Harrison *et al.*, 2009). To study the combined influence of the two haemoglobin genes (*hpuAB* and *hmbR*) on meningococcal disease and carriage, the distribution of both receptors was investigated using four unrelated isolate collections containing 305 and 214 meningococcal isolates from carriage samples and disease cases, respectively. The majority (*n* = 422) represented nine serogroups (A, B, C, 29E, H, W-135, X, Y and Z), while 97 were non-serogroupable. A total of 33 CCs were represented, with most isolates belonging to 16 CCs.

Overall, we observed that 47 % of isolates had both genes, while 28 % had *hmbR* alone, and 21 % had *hpuAB* as their sole haemoglobin receptor. A minority (4 %) lacked both systems, and most of these (91 %) were obtained from carriers. Comparisons were made between the presence of both genes versus one gene and between *hmbR* only and *hpuAB* only (Table 1). No significant difference was observed in the frequencies of both genes versus *hmbR* only between disease and carriage isolates. In contrast, both genes or *hmbR* only occurred at significantly higher frequencies than *hpuAB* only in disease isolates. These differences were obtained for each isolate collection separately as well as in combination. An over-representation of *hpuAB* in carriage versus disease isolates was evident, although the significance was reduced on exclusion of the 2008 carriage study (Table 2), which includes multiple isolates resulting from clonal expansion of some strains (Bidmos *et al.*, 2011).

**Table 1.** Distribution of haemoglobin receptors in disease and carriage isolates

Strain collection	Group	Both present	*hmbR* only	*hpuAB* only	Both absent	Total	Disease association OR (95 % CI)		
							Both vs *hpuAB*	Both vs *hmbR*	*hmbR* vs *hpuAB*
All	Disease	113 (52 %)	87 (41 %)	12 (6 %)	2 (1 %)	214	6.83 (3.56–13.0)*	0.58 (0.38–0.87)†	11.88 (5.98–23.59)*
	Carriage	131 (43 %)	58 (19 %)	95 (31 %)	21 (7 %)	305			
All minus 2008	Disease	113 (52 %)	87 (41 %)	12 (6 %)	2 (1 %)	214	4.85 (2.44–9.64)*	0.67 (0.42–1.02)‡	7.4 (3.6–15.21)*
	Carriage	97 (45 %)	49 (22 %)	50 (23 %)	21 (10 %)	217			
Czech 1993	Disease	26 (68 %)	9 (24 %)	1 (3 %)	2 (5 %)	38	10.2 (1.3–80.29)	1.47 (0.6–3.6)	6.92 (0.81–59.27)
	Carriage	51 (44 %)	26 (23 %)	20 (17 %)	18 (16 %)	115			
107 MLST isolates	Disease	47 (51 %)	33 (37 %)	10 (12 %)	0	90	3.92 (1.0–15.4)	0.95 (0.25–3.63)	4.13 (0.93–18.37)
	Carriage	6 (35 %)	4 (24 %)	5 (29 %)	2 (12 %)	17			
UK 1999	Disease	39 (51 %)	37 (48 %)	1 (1 %)	0	77	25.0 (3.23–193.8)	0.49 (0.24–1.0)	51.39 (6.44–410.2)
	Carriage	39 (48 %)	18 (22 %)	25 (30 %)	0	82			
ST-41/44	Disease	4 (9 %)	40 (89 %)	0	1 (2 %)	45	−	0.47 (0.1–2.28)	−
	Carriage	3 (9 %)	13 (38 %)	0	18 (53 %)	34			

* *P* <0.0001.

† *P* = 0.02.

‡ Not significant.

**Table 2.** Distribution of *hpuAB* in disease and carriage isolates

Strain collection	Group	*hpuAB*-positive	*hpuAB*-negative	Total	Disease association OR (95 % CI)	Carriage association OR (95 % CI)
All	Disease	125 (58 %)	89 (42 %)	214	0.49 (0.38–0.71)	2.04 (1.4–2.96)*
	Carriage	226 (74 %)	79 (26 %)	305		
All minus 2008	Disease	125 (58 %)	89 (42 %)	214	0.67 (0.45–0.995)	1.50 (1.01–2.22)†
	Carriage	147 (68 %)	70 (32 %)	217		

* *P* = 0.0002.

† *P* = 0.047.

Serogroups A, C and 29E exhibited a high prevalence of these receptors, with 75, 87 and 57 %, respectively, of strains from these serogroups harbouring both genes. Some serogroups exhibited an under-representation of the *hmbR*-only phenotype; thus, none of the isolates from serogroup Y and only 2 % of 29E serogroupable strains had this receptor as the sole haemoglobin-binding protein. In contrast, *hmbR* only was present in 59 % of serogroup B strains, with only 12 % having an *hpuAB*-only phenotype. There was no significant difference between the presence of both genes versus *hmbR* only in disease versus carriage serogroup B isolates (OR 0.58, 0.294–1.136 at 95 % CI, *P* = 0.12).

A bias in the distribution of haemoglobin receptors between CCs was also evident (Table 3). One group of CCs had a high prevalence of *hmbR*-only isolates (e.g. 92, 90, 67 and 63 % of isolates from ST-18, ST-32, ST-41/44 and ST-269, respectively). Other CCs exhibited a high level of both receptors (e.g. 98, 92 and 90 % of isolates from ST-11, ST-5 and ST-8, respectively). In contrast, ST-167, ST-1 and ST-22 had high levels of *hpuAB*-only (91, 62 and 61 %, respectively) and low levels of *hmbR*-only isolates, while 100 % of isolates analysed from ST-174, ST-106 and ST-23 had *hpuAB*-only phenotypes. None of the isolates from the ST-41/44 complex had *hpuAB* as a sole haemoglobin receptor, while 53 % of carriage isolates from this CC lacked both receptors.

**Table 3.** Distribution of *hpuAB* deletion mechanism and haemoglobin receptors by CC

CC	Number of isolates	*hpuAB* deletion mechanism		Receptor status				Disease : carriage ratio*
		IS element	Complete deletion	Both present	*hmbR* only	*hpuAB* only	Both absent	
**Group 1: HmbR only in 10–100 % of isolates**								
ST-41/44	79	69	3	7	53	0	19	1.2
ST-18	13	12	0	1	12	0	0	5.5
ST-213	8	6	0	2	6	0	0	0.6
ST-461	1	1	0	0	1	0	0	−
ST-334	2	1	0	1	1	0	0	−
ST-32	21	0	19	2	19	0	0	3.5
ST-269	19	0	12	7	12	0	0	2.8
ST-35	4	0	3	1	3	0	0	0.5
Unspecified	36	33	3	0	32	0	4	−
**Group 2: both receptors in >90 % isolates**								
ST-11	59	1	0	58	1	0	0	6.6
ST-5	12	1	0	11	1	0	0	19.5
ST-4	11	0	0	11	0	0	0	−
ST-92	15	0	0	15	0	0	0	−
ST-8	10	0	1	9	1	0	0	24.5
ST-116	6	0	0	6	0	0	0	−
ST-231	3	0	0	3	0	0	0	−
ST-37	2	0	0	2	0	0	0	−
ST-53	1	0	0	1	0	0	0	−
ST-162	1	0	0	1	0	0	0	−
ST-292	1	0	0	1	0	0	0	−
ST-364	1	0	0	1	0	0	0	−
ST-750	1	0	0	1	0	0	0	−
Unspecified	61	0	0	61	0	0	0	−
**Group 3: HpuAB only in 10–90 % of isolates**								
ST-60	29	0	1	18	1	10	0	0.7
ST-1157	10	0	0	8	0	2	0	−
ST-198	5	0	0	2	0	3	0	<0.1
ST-1	13	0	1	4	1	8	0	5.5
ST-22	18	0	0	7	0	11	0	0.6
ST-103	5	1	0	2	1	2	0	1.2
**Group 4: HpuAB only in >90 % of isolates**								
ST-174	15	0	0	0	0	15	0	−
ST-106	11	0	0	0	0	11	0	−
ST-23	13	0	0	0	0	13	0	0.8
ST-167	11	0	0	1	0	10	0	0.5
ST-254	1	0	0	0	0	1	0	−
ST-549	1	0	0	0	0	1	0	−
Unspecified	20	0	0	0	0	20	0	−

* Disease : carriage ratios were taken from Caugant & Maiden (2009).

### Genetic arrangement of the *hpuAB* locus and deletion mechanisms

**Fig. 1.**
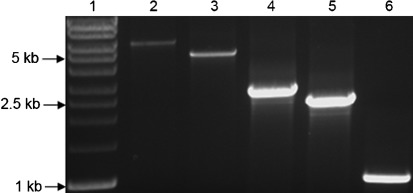
Variations in the size of the *hpuAB* locus among *N. meningitidis* isolates. The *hpuAB* locus was amplified with primers specific for flanking genes. Amplicons were grouped into five classes based on size, and representative samples are shown. Lanes: 1, size standards; 2, Hpu1, *hpu+* strain (Z2491); 3, Hpu2, *hpu+* strain (Z6414); 4, ΔHpu3, *hpu*− strain (Z4686); 5, ΔHpu4, *hpu*− strain (Z6427); 6, ΔHpu5, *hpu*− strain (Z4685).

The arrangement and deletion mechanisms for the *hpuAB* locus were elucidated by amplification of the regions flanking this locus using primers specific for adjacent genes (encoding a hypothetical periplasmic protein and GroEL, a chaperone). These primers are predicted to produce an ~5.6 kb fragment from meningococcal strains with a full-length *hpuAB* system. This analysis revealed a variation in the size of PCR products from isolates that had previously tested positive for this system (Fig. 1, lanes 2 and 3), and amplicons (Fig. 1, lanes 4, 5 and 6) significantly smaller than those of the reference strain (Z2491) for *hpuAB*-negative isolates. A total of 264 (96 from *hpuAB*-positive and 168 from *hpuAB*-deleted strains) amplicons were generated and divided into five groups based on size and the presence (Hpu) or absence (ΔHpu) of the receptor (Fig. 1), with the following numbers of isolates in each group: Hpu1, 30; Hpu2, 66; ΔHpu3, 74; ΔHpu4, 51; and ΔHpu5, 43 (data not shown).

**Fig. 2.**
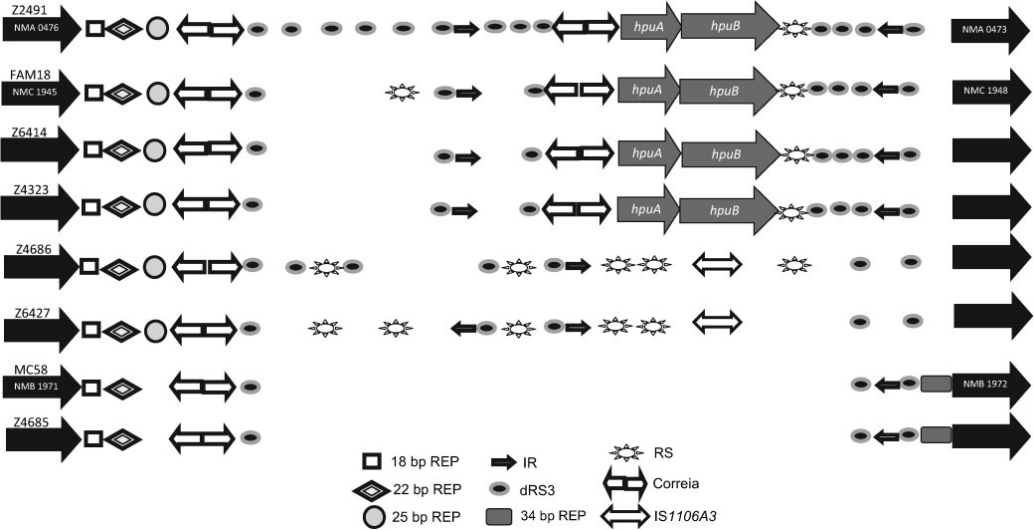
Genetic arrangement of the *hpuAB* locus in *N. meningitidis* isolates. Directional arrows represent ORFs. Repetitive elements and the IS element are represented by different symbols. An absence of symbols indicates a region that is deleted in a particular clone. Note that the figure is not drawn to scale but is a representation of the various elements and genes present in this locus.

To identify the mechanism responsible for the PCR product size variation, representative isolates of each group were sequenced and compared with the sequence of reference strain Z2491. A cluster of nine dRS3 (ATTCCCN8-GGGAAT) repetitive elements is present upstream of *hpuA* in the reference strain. This cluster is flanked by Correia elements and can be further subdivided into two direct repeats of 118 bp. In addition, REP elements of variable lengths (18, 22 and 25 bp) are also present. The analysis of strains positive for *hpuAB* (4323 and 6414), but with smaller amplicons (Hpu2) than Z2491, demonstrated a deletion of 600 bp in an upstream sequence flanked by Correia elements (Fig. 2). This deletion can be explained by a recombination event between dRS3 sequences in the upstream region.

To explain the deletion mechanism for *hpuAB*, flanking regions were amplified with nested PCR primers for chromosomal walking. Subsequent sequencing of the products detected a variable number of dRS3 repeats in each of the deletion types. In addition, strains 4686 and 6427 (ΔHpu3 and ΔHpu4, respectively) contained a sequence with a high identity (95 %) to the published sequence of the transposase of IS*1106A3*. The complete deletion of the system in two other Hpu-negative strains, MC58 and 4685 (ΔHpu5), appears to have occurred via recombination between homologous dRS3 sequences in upstream and downstream regions, leaving a 1 kb fragment composed of repetitive elements. The presence of IS elements in non-sequenced strains with ΔHpu3 and ΔHpu4 deletion types was confirmed by performing a PCR with a flanking primer (hpu-rev13) and a primer (hpuF-seq2) specific for the IS element (data not shown).

### Distribution of *hpuAB* deletion mechanisms within CCs

Phylogenetic analyses revealed that the majority of *hpuAB*-negative isolates (79 %) were restricted to 14 CCs (ST-41/44, ST-11, ST-60, ST-32, ST-269, ST-1, ST-18, ST-5, ST-8, ST-213, ST-35, ST-103, ST-334 and ST-461) (Table 3). For the 125 *hpuAB*-negative isolates containing the IS element, 55 % belonged to the ST41/44 complex, while 10 % were from ST-18 with high dissemination within these CCs and in ST-213 (75–92 % of isolates from each complex). Similarly, 72 % of the 43 isolates with a complete deletion of *hpuAB* were from two CCs, 44 % from ST-32 and 28 % from ST-269, with 75–90 % of isolates from these CCs containing the deletion.

### PV status of the receptors in disease and carriage strains

**Fig. 3.**
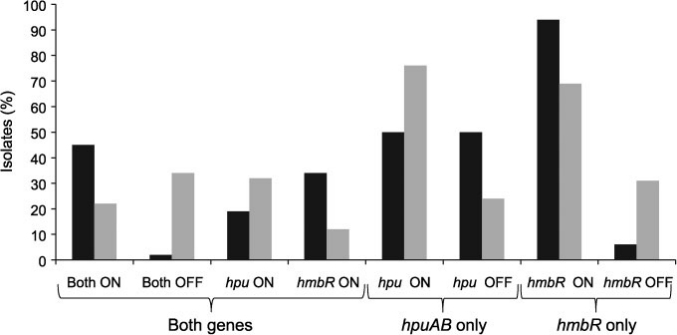
PV status of haemoglobin receptors in meningococcal disease and carriage isolates. PV status for the *hmbR* and *hpuAB* (*hpu*) genes was determined based on the length of the repeat tract. This tract is located within the reading frame such that alterations in these tracts switch the genes ON and OFF. Disease (*n* = 90) and carriage (*n* = 103) isolates were separated into three groups based on presence/absence of the receptors: both receptors present (*n* = 47 and 41 for disease and carriage, respectively); *hpuAB* only (*n* = 10 and 54); and *hmbR* only (*n* = 33 and 13). Strains with both receptors were separated into four groups depending on the ON/OFF status of the receptors. Strains with a single receptor will exhibit either an ON or OFF phenotype and so fall into two groups. Black bars, disease isolates; grey bars, carriage isolates.

The ON/OFF status of the receptors was examined in two isolate collections either by sequencing PCR products or by a combination of sizing of fluorescent PCR products (i.e GeneScan) and sequencing (Fig. 3). The 107 MLST isolates comprised strains representative of the major meningococcal clones causing disease in the latter half of the 20th century. The 2008/2009 carriage group was chosen in order to examine how expression of the haemoglobin receptors was influenced by carriage and because these isolates were subject to only one passage *in vitro*. Minimal passage combined with the low PV frequencies (~1×10^−4^) of meningococcal genes (Martin *et al.*, 2004) reduced the chances of isolating phase variants during passage of single colonies. The observation of both ON and OFF variants amongst the carriage isolates indicated that *in vitro* growth did not select for particular expression states of these receptors. For disease isolates harbouring both genes (47 isolates), the majority (99 %) had either both (45 %) or one gene (54 %) in an ON state. In contrast, for carriage isolates with both receptors (41 isolates), only 22 % had both receptors ON and 34 % had both OFF. For *hmbR*-only strains, the receptor was ON in 94 % and 69 % of disease and carriage isolates, respectively. Surprisingly, in *hpuAB*-only strains, the receptor was ON in 75 % of carriage isolates but only 50 % of disease isolates, although this latter figure is based on analysis of a small number of samples. Overall, 91 % (82/90) of disease and 71 % (73/103) of carriage isolates were found to have at least one haemoglobin receptor in an ON state (OR 4.21, 95 % CI 1.82–9.77, *P* = 0.0005).

**Fig. 4.**
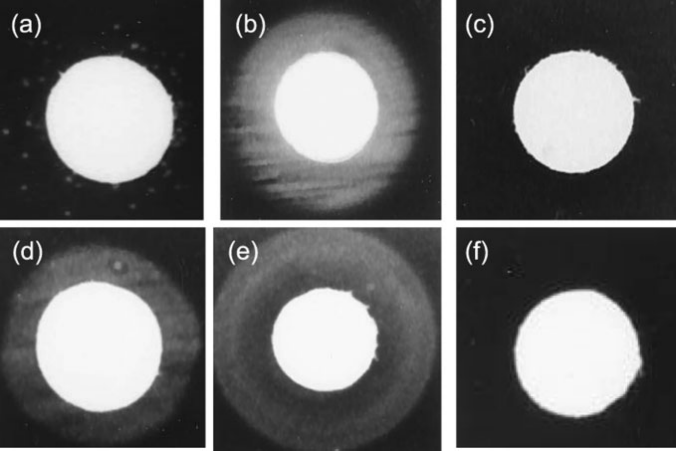
Phenotypic analysis of *hmbR* and *hpuAB* phase variants. An *hpuAB* OFF variant of strain N54 (this strain lacks the *hmbR* gene) and an *hmbR* ON variant of strain MC58 (lacks the *hpuAB* genes) were tested for growth with haemoglobin or transferrin as the sole iron source. Suspensions of meningococcal strains were seeded onto MHA plates containing 40 µM desferal. Disks were inoculated with 10 µl of either human haemoglobin (10 µg ml^−1^) or transferrin (50 mg ml^−1^), or were uninoculated. Growth was recorded after overnight growth. (a) N54, with haemoglobin; (b) N54, with transferrin; (c) N54, no iron; (d) MC58, with haemoglobin; (e) MC58, with transferrin; (f) MC58, no iron.

The expression status of the haemoglobin receptors of a representative sample of these strains was confirmed by testing for the ability to utilize haemoglobin as the only iron source. Strain N54 had a single receptor, HpuAB, in the OFF state and only exhibited confluent growth in the presence of transferrin (Fig. 4a–c). Scattered colonies around the haemoglobin-containing disc represented a small number of ON phase variants (Fig. 4a). In contrast, strain MC58 had a single receptor, HmbR, in the ON state and exhibited confluent growth in the presence of haemoglobin and transferrin, although not without an added iron source (Fig. 4d–f). Similar results were obtained for eight further strains, indicating that the genetic screen for ON/OFF PV states correlates with phenotypic expression of the haemoglobin receptors.

### Tract length distributions of haemoglobin receptors

**Fig. 5.**
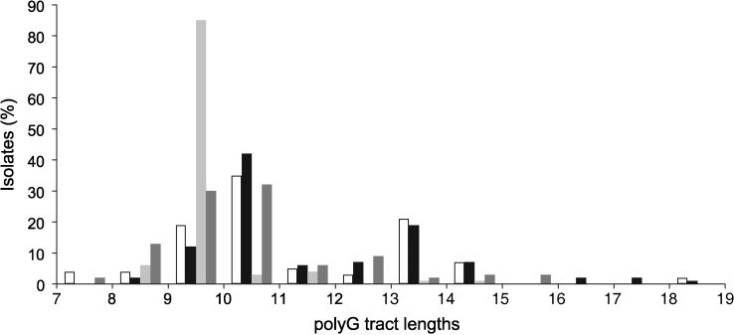
Tract distribution of *hmbR* and *hpuAB* in meningococcal disease and carriage isolates. Lengths of the polyG tracts of *hmbR* and *hpuAB* were determined by amplification with specific primers and by DNA sequencing of the products. White bars, *hpuAB* in disease isolates; black bars, *hpuAB* in carriage isolates; light-grey bars, *hmbR* in disease isolates; dark-grey bars, *hmbR* in carriage isolates. An ON PV state is produced by 9G, 12G, 15G and 18G for HmbR, and by 7G, 10G, 13G, 16G and 19G for HpuAB.

The length of the homopolymer repeat tracts is one of the key factors in controlling the PV rate (De Bolle *et al.*, 2000; Richardson *et al.*, 2002). The sequences for *hpu*/*hmbR* were compared for variations in the polyG repeat tracts (Fig. 5). For *hpuA*, tract lengths in both groups ranged from 7G to 19G, with a modal repeat number of 10G, an ON number of repeats. A significant minority of isolates possessed 13G, which is also an ON state for this gene. The repeat tract of *hmbR* varied between 7G and 15G. The modal repeat numbers for the 107 MLST isolates and 2008/2009 carriage strains were 9G and 10G, respectively, reflecting differences in the proportion of isolates with this gene in an ON state (i.e. 9G is ON). Tract lengths longer than the modal numbers were observed at significantly higher frequencies as compared with shorter lengths for both genes (*P*<0.05; data not shown).

## Discussion

Meningococci express a variety of iron-acquisition systems on their surfaces, enabling acquisition of iron from a range of host complexes, including transferrin, lactoferrin and haem-containing proteins (Perkins-Balding *et al.*, 2004). The relative importance of each of these sources for invasive disease or carriage is still unclear. HmbR and HpuAB are surface receptors involved in acquisition of iron from haemoglobin or haemoglobin–haptoglobin complexes and undergo PV due to polyG repeat tracts present in the reading frame. Richardson & Stojiljkovic (1999) analysed a limited number of clinical samples and found that >50 % harbour both haemoglobin receptors, suggesting the importance of these receptors for invasion. Subsequently, HmbR was shown to be over-represented in disease isolates and to exhibit significant levels of antigenic variation, suggesting that this receptor facilitates invasion or dissemination within the body but is also subject to immune selection (Evans *et al.*, 2010; Harrison *et al.*, 2009). In the present study, the antigenic variation, distribution and PV of both haemoglobin receptors were assessed in four different and unrelated isolate collections. A complex relationship between presence/expression of these receptors and meningococcal disease and carriage states was revealed.

Antigenic variation of cell surface structures in bacterial pathogens is associated with escape from host defence systems and has implications for effective vaccine development. Alignment of the nucleotide sequences of the complete *hpuA* sequences from eight isolates and of partial sequences from several other strains detected regions of significant variation interspersed with conserved regions and an uneven distribution of single nucleotide polymorphisms. A similar pattern of variation has been observed in several other surface-exposed meningococcal proteins whose variable regions are known targets of host immune responses (Russell *et al.*, 2004; Thompson *et al.*, 2003). There are currently no structural or functional predictions for HpuA. Thus it is unclear whether these polymorphisms can be attributed to surface-exposed loops; however, it is likely that this variation is evidence of a high level of antigenic variation of HpuA and has arisen due to immune selection. A more detailed analysis of the diversity of both *hpuA* and *hpuB* is required to determine whether antigenic variants of this receptor are subject to CC structuring, as seen for *hmbR*, *porA* and *fetA* (Evans *et al.*, 2010; Russell *et al.*, 2004; Thompson *et al.*, 2003).

Investigation of the distribution of genes in disease and carriage isolates is an important tool in the hunt for new virulence factors and may lead to an improved understanding of microbial pathogenesis and disease progression. A significant under-representation of an *hpuAB*-only phenotype (Table 1) was observed in disease isolates as compared with both receptors and an *hmbR*-only phenotype. This distribution may have arisen due to a combination of selection against HpuAB and a requirement for haemoglobin acquisition during invasive disease. Thus, HpuAB may be more immunogenic than HmbR, resulting in stronger selection against HpuAB-only strains than against HmbR-only strains during invasive disease and low levels of selection against strains with both receptors, as in this case switching HpuAB OFF and HmbR ON enables escape from immunity and maintenance of haemoglobin acquisition. This immunological pressure would be less severe during carriage in the nasopharynx, as HpuAB-only strains could switch the receptor OFF and acquire iron through another receptor such as the lactoferrin-binding protein. Intriguingly, all CCs with high disease : carriage ratios (>6) have both receptors (Table 3), suggesting an additional advantage associated with both receptors which may be connected with a propensity to cause disease or the rapid transmission required to compensate for low levels of carriage.

An alternative explanation is that the differing substrate specificities and affinities of these receptors influence their distribution in disease versus carriage isolates. Haemoglobin–haptoglobin complexes are likely to be a major source of iron in the nasopharynx and during initial exposure to serum, whereas free haemoglobin is likely to be present only at low levels. As haemoglobin–haptoglobin complexes can only be utilized via the HpuAB receptor, this specificity might imply that an Hpu+ phenotype would be advantageous during carriage and result in over-representation of this receptor among carriage isolates. This seems unlikely, as *hmbR*-only strains are prevalent in carriers, suggesting that free haemoglobin is available during carriage. It is also unclear why this specificity would have resulted in the low prevalence of an *hpu*-only phenotype among disease isolates, as haptoglobin–haemoglobin complexes should be readily available during systemic spread. Conversely, over-representation of *hmbR* in disease isolates may be due to this receptor having a higher affinity for haemoglobin than HpuAB and a greater availability of free haemoglobin during disease due to tissue destruction and cell lysis. Recently, growth of one meningococcal strain, MC58 (an *hmbR*-only strain), with haemoglobin but not transferrin as the sole iron source has been associated with induction of virulence genes, suggesting a direct link between haemoglobin utilization and a pathogenic phenotype (Jordan & Saunders, 2009). Thus the release of free haemoglobin by the action of haemolysins and other toxins would not only enhance growth of *hmbR+* strains but also induce the expression of virulence-associated genes, leading to an increase in the likelihood of these strains causing invasive disease. If HmbR facilitates growth under these conditions, this would predict an over-representation of strains with both receptors or with an *hmbR*-only phenotype among disease isolates, as observed herein. Finally, the absence of both receptors in some carriage isolates implies either that acquisition of iron or haem from haemoglobin is not essential for persistence in the human nasopharynx or that an alternative haemoglobin receptor is present.

A variety and abundance of repetitive elements is evident in neisserial genomes, and repetitive elements are associated with the deletion of surface determinants such as FetA and PorA (Claus *et al.*, 1997; Marsh *et al.*, 2007; van der Ende *et al.*, 1999). Deletion of *hpuAB* was found to be due to replacement by an IS element or recombination between repetitive elements (Fig. 2). Although present in many strains, only three types of deletion events covered all *hpuAB*-negative isolates (Fig. 3), and each ST was associated with a specific type of deletion event (Table 3). Stable lineages of this type suggest that deletions are infrequent, possibly because the selective advantage associated with deletion of these genes is low and may have required acquisition of *hmbR* as a functional replacement before dissemination of the deletion could occur.

PV of the haemoglobin receptors is likely to have a significant impact on immune evasion and niche adaptation by meningococci. Richardson *et al.* (2002) demonstrated that high rates of PV of these receptors were associated with epidemic spread of meningococci. This suggests that stochastic changes in expression facilitate immune evasion and transmission once the host population has developed herd immunity to circulating meningococcal strains. The presence of a significantly higher number of isolates with one or both genes in an ON state for disease (91 %) as compared with carriage (71 %) isolates reinforces the view that haemoglobin accumulation is required during invasive disease. Furthermore, the observation of a significant number of isolates with their haemoglobin receptors in the OFF state during carriage implies a less stringent requirement for haemoglobin acquisition during carriage and more frequent immune evasion. Examination of specific immune responses to these receptors will be required to determine whether PV status is influenced by adaptive immune responses.

A comment on the potential biases in analysis of PV states is appropriate at this point. As noted in Results, minimal passage, low PV frequencies (<1×10^−4^; Lewis *et al.*, 1999; Richardson & Stojiljkovic, 1999), similar ON-to-OFF/OFF-to-ON switching rates and lack of selection during *in vitro* growth reduced the likelihood of switches occurring during the growth and analysis of isolates, such that the overall findings are likely to provide a strong reflection of the *in vivo* PV states of these loci. Single colony analysis, as used herein, may not reflect situations in carriers or patients who are colonized or infected with ratios of ON and OFF phase variants of <3 : 1. This situation can only be overcome by analysis of multiple colony isolates or direct analysis of bacterial DNA without growth to detect the prevalent PV state. Preliminary analyses of multiple colonies from carriers detected ratios for ON-to-OFF/OFF-to-ON variants of <3 : 1 in two of 12 individuals (S. B. Redkar and others, unpublished data), but further studies are required to ascertain how frequent such low ratios of variants are in carriers and patients. It seems unlikely, however, that we would have observed such a high prevalence of ON phenotypes if low ratios of variants were present in a significant number of carriers or patients.

Acquisition of iron is considered a prerequisite for bacterial disease. The observation of selection against an HpuAB-only phenotype in meningococcal disease isolates indicates that not all iron-acquisition systems may facilitate bacterial pathogenesis. Contrastingly, disease isolates have at least one receptor, either HpuAB or HmbR, in an ON PV state. Thus acquisition of haemoglobin appears to facilitate invasive meningococcal disease and is mediated by either a combination of haemoglobin receptors or HmbR alone. Further work is required to determine whether the high prevalence of *hpuAB*-negative variants and PV of these systems are driven by immune evasion, and whether expression of these receptors is required for meningococcal disease.

## Supplementary Material

Supplementary Materials
