# Supplementary material for: Influence of the combination and phase variation status of the haemoglobin receptors HmbR and HpuAB on meningococcal virulence
Source: Microbiology (Reading). 2011 May;157(Pt 5):1446–56. doi: 10.1099/mic.0.046946-0 (PMC3352162; doi:10.1099/mic.0.046946-0)
Supplement: Supplementary Materials [file supp_157_5_1446__index.html]

Influence of the combination and phase variation status of the haemoglobin receptors HmbR and HpuAB on meningococcal virulence — Supplementary data 

# Influence of the combination and phase variation status of the haemoglobin receptors HmbR and HpuAB on meningococcal virulence

## Supplementary data

### Influence of the combination and phase variation status of the haemoglobin receptors HmbR and HpuAB on meningococcal virulence, by I. Tauseef, O. B. Harrison, K. G. Wooldridge, I. M. Feavers, K. R. Neal, S. J. Gray, P. Kriz, D. P. J. Turner, D. A. A. Ala�Aldeen, M. C. J. Maiden and C. D. Bayliss

*Microbiology* vol. **157**, part 5, pp. 1446 - 1456

**Supplementary Fig. S1 legend**  [PDF]  (17 KB)

**Supplementary Fig. S1.** Alignment of the amino acid sequences of HpuA from carriage and disease isolates of *Neisseria meningitidis* [PDF]  (49 KB)
