# Supplementary material for: Influence of the combination and phase variation status of the haemoglobin receptors HmbR and HpuAB on meningococcal virulence
Source: Microbiology (Reading). 2011 May;157(Pt 5):1446–56. doi: 10.1099/mic.0.046946-0 (PMC3352162; doi:10.1099/mic.0.046946-0)
Supplement: Supplementary Materials [file supp_157.5.1446_mic046946figS1.pdf]

```
              *        20         *        40         *        60         *        80         *       100
N222hpuA  : VAEPHVPVSIPTATPL-TGEVKLSSDNSKIENINTANTETHPPRRTRRSLYASPQNTSSGISIQQREVEKIYFGVKSPEKSFIFQTPGGAQYALSSYADPIVPSYSS : 106
Z2491hpuA : VAEPHVPVSIPTATPL-TGEVKLSSDNSKIENINTANTETHPPRRTRRSLYASPQNTSSGISIQQREVEKIYFGVKSPEKSFIFQTPGGAQYALSSYADPIVPSYSS : 106
N117hpuA  : VAEPHVPVSIPTATPL-TGEVKLSSDNSKIENINTANTETHPPRRTRRSLYASPQNTSSGISIQQREVEKIYFGVKSPEKSFIFQTPGGAQYALSSYADPIVPSYSS : 106
N119hpuA  : VAEPHVPVSIPTATPLPAGEVTLSDDSVNIVNINTANTETHPPRRTRRSLYASPQNTSAGISIQQREVEKDYFGYKSKETSFIFQTPGGAQYALSSYSDPIVPSYSS : 107
N134hpuA  : VAEPHVPVSIPTATPLPAGEVTLSDDSVNIVNINTANTETHPPRRTRRSLYASPQNTSAGISIQQREVEKDYFGYKSKETSFIFQTPGGAQYALSSYSDPIVPSYSS : 107
N114hpuA  : VAEPHVPVSIPTATPLPAGEVTLSDDSVNIVNINTANTETHPPRRTRRSLYASPQNTSAGISIQQREVEKDYFGYKSKETSFIFQTPGGAQYALSSYSDPIVPSYSS : 107
N88hpuA   : VAEPHVPVSIPTATPLP-GEVTLSDDSVNIVNINTAGTG----------------STSSGISIQQRKYNVTSYGFTREEKAFIFKTPGGAQYTLSSYADPIVPSYSS :  90
N138hpuA  : VAEPHVPVSIPTATPLP-GEVTLSDDSVNIVNINTAGTG----------------STSSGISIQQRKYNVTSYGFTREEKAFIFKTPGGAQYTLSSYADPIVPSYSS :  90
8047hpuA  : VAEPHVPVSIPTATPL-TGEVKLTDDNSKIENINTANTG----------------NTS-GISIQQREYKVNNYGVESTAKAFIFKTPGGAQYTLSSYADPIVPSYSS :  89
Fam18hpuA : VAEPHVPVSIPTATPLPTGEVKLSDDNSKIENINTADTG----------------STS-GISIQQRKYEVESYGIKREEKAFIFQTPGGAQYALSSYADPITVSYSS :  90

               *       120         *       140         *       160         *       180         *       200         *
N222hpuA  : PDFKIPDRHAGQRLADGSRIFICCSDSGATSYAEITKQDYMK--FGAWIGPNGEIDLFAGGFPVGKTPPPAFSYGNSTPETALSKGKITYQVWGIRVKDGQFVTSSY : 211
Z2491hpuA : PDFKIPDRHAGQRLADGSRIFICCSDSGATSYAEITKQDYMETKFGAWIGPNGEIDLFAGGFPIGKTPPPAFSWGSPTTETALSKGKITYQVWGIRVRNGQFVTSSY : 213
N117hpuA  : PDFKIPDRHAGQRLADGSRIFICCSDSGATSYAEITKQDYMK--FGAWIGPNGEIDLFAGGFPIGKTPPPAFSWGSPTTETALSKGKITYQVWGIRVRNGQFVTSSY : 211
N119hpuA  : PDFKIPDRHAGQRLADGSRIFICCSDSGATTYAEITKQDYMK--FGAWIGPNGEIDLFAGGFPVGKTPKPAYSWGDDTPETAG-KGKITYQVWGIRVKDGQFVTSSY : 211
N134hpuA  : PDFKIPDRHAGQRLADGSRIFICCSDSGASTYAEITKQDYMK--FGAWIGPNGEIDLFAGGFPVGKTPKPAYSWGDDTPETAG-KGKITYQVWGIRVKDGQFVTSSY : 211
N114hpuA  : PDFKIPDRHAGQRLADGSRIFICCSDSGATTYAEITKQDYMK--FGAWIGPNGEIDLFAGGFPVGKTPKPAYSWGDDTPETAG-KGKITYQVWGIRVKDGQFVTSSY : 211
N88hpuA   : PDFKIPDRHAGQRLADGSRIFICCSDSGATNQAEITKQDYMETKFGAWIGPNGEIDLFAGGFPVGKTPASSSYYS-STLETAG-KGKITYQVWGIRVKDGQFVTSSY : 195
N138hpuA  : PDFKIPDRHAGQRLADGSRIFICCSDSGATNQAEITKQDYMK--FGAWIGPNGEIDLFAGGFPVGKTPASSSYYS-STLETAG-KGKITYQVWGIRVKDGQFVTSSY : 193
8047hpuA  : PDFKIPDRHAGQRLADGSRIFICCSDSGATNQAEITKQDYMETKFGAWIGPNGEIDLFAGGFPVGKTPASSSYYGSSTPETQG-KGKITYQVWGIRVRNGQFVTSSY : 195
Fam18hpuA : PDFKIPDRHAGQRLADGSRIFICCSESGATSYAEITKQDYMETKFGAWIGPNGEIDLFAGGFPVGKTPKPKYSWGNTEPETKG-KGKITYQVWGIRVRNRQFVTSSY : 196

               220         *       240         *       260         *       280         *       300         *       320
N222hpuA  : TPPKGSSFTG--YTNTPVLSFITANFNSNKLAGEIRGNSDYGPSVKIENATISGPSFSGNATSGGKTGNLEGKFFGKFNGSYGNTETSIGGKITFKDDRSLDTVFGG : 316
Z2491hpuA : TPPKGSSWSG--YQNTPVLSFITANFNSNKLAGEIIGNSDYGPNVKIENATIDGLSFSGDATSNGKTGNLEGKFFGKFN-STRSSDTGIGGKITFKDDRSLDTVFGG : 317
N117hpuA  : TPPKGSSWSG--YQNTPVLSFITANFNSNKLAGEIRGNSDYGPDVEIKEAQIDGLSFSGDATSGGKTGKLEGKFFGKFN-GYRDTETSIGGKITFDGDRSLDTVFGG : 315
N119hpuA  : TPPKGSSFTG--YTNTPVLSFITANFNSNKLAGKIIGNSDYGPDVEIKEAQIDGLSFSGDATSGGKTGKLEGKFFGKFDSSYDR-DTSIGGKITFDGDRSLDTVFGG : 315
N134hpuA  : TPPKGSSFTG--YTNTPVLSFITANFNSNKLAGKIIGNSDYGPDVEIKEAQIDGLSFSGDATSGGKTGKLEGKFFGKFDSSYDR-DTSIGGKITFDGDRSLDTVFGG : 315
N114hpuA  : TPPKGSSFTG--YTNTPVLSFITANFNSNKLAGKIIGNSDYGPDVEIKEAQIDGLSFSGDATSGGKTGKLEGKFFGKFDSSYDR-DTSIGGKITFDGDRSLDTVFGG : 315
N88hpuA   : TPPKNSSSYLYKPTNTPVLSFITANFNSNKLAGEIIGNSDYGPDVKIENATIDGLSFSGNATSGGKTGKLDGKFFGKFDSTRSS-DTGIGGKITFKDDRSLDTVFGG : 301
N138hpuA  : TPPKNSSSYLYKPTNTPVLSFITANFNSNKLAGEIIGNSDYGPDVKIENATIDGLSFSGNATSGGKTGKLDGKFFGKFDSTRSS-DTGIGGKITFKDDRSLDTVFGG : 299
8047hpuA  : TPPKNSGYYSSTPTNTPVLSFITANFNTHKLSGEIIGNSDYGPNVKIENATIDGLSFSGDATSGGKNGKLEGKFFGKFNGYRDT-ETSIGGKITFDGDRSLDTVFGG : 301
Fam18hpuA : TPPKSGSYYG-TLANTPVLSFITANFNSNKLAGEIRGNSDYGPSVKIENATINSLSFSGTATSGGKTGNLEGKFFGKFNGYRDT-ETSIGGKITFDGDRSLDTVFGG : 301

                  *       340
N222hpuA  : VSYVKKLDETANRDTEHLTKQ : 337
Z2491hpuA : VIYEKKLDDKTSQDTNHLKK- : 337
N117hpuA  : VSYKKELENNTDTSTTHLTK- : 335
N119hpuA  : VSYKKELENNTDMSTTHLTK- : 335
N134hpuA  : VSYKKELENNTDMSTTHLTK- : 335
N114hpuA  : VSYKKELENNTDMSTTHLTK- : 335
N88hpuA   : VIYEKKLDDKTSQDTTHLTK- : 321
N138hpuA  : VIYEKKLDDKTSQDTTHLTK- : 319
```

```
8047hpuA  : VSYKKELENNTDTSTTHLTK- : 321
Fam18hpuA : VSYKKELENNTDTSTTHLTK- : 321
```

Influence of the combination and phase variation status of the haemoglobin receptors HmbR and HpuAB on meningococcal virulence

By: Isfahan Tauseef, Odile B. Harrison, Karl G. Wooldridge, Ian M. Feavers, Keith R. Neal, Stephen J. Gray, Paula Kriz, David P. J. Turner, Dlawer A. A. Ala'Aldeen, Martin C. J. Maiden and Christopher D. Bayliss

Supplementary Fig. S1
