# Supplementary material for: Influence of the combination and phase variation status of the haemoglobin receptors HmbR and HpuAB on meningococcal virulence
Source: Microbiology (Reading). 2011 May;157(Pt 5):1446–56. doi: 10.1099/mic.0.046946-0 (PMC3352162; doi:10.1099/mic.0.046946-0)
Supplement: Supplementary Materials [file supp_157.5.1446_mic046946figS1_legend.pdf]

Influence of the combination and phase variation status of the haemoglobin receptors HmbR and HpuAB on meningococcal virulence

By: Isfahan Tauseef, Odile B. Harrison, Karl G. Wooldridge, Ian M. Feavers, Keith R. Neal, Stephen J. Gray, Paula Kriz, David P. J. Turner, Dlawer A. A. Ala'Aldeen, Martin C. J. Maiden and Christopher D. Bayliss

SUPPLEMENTARY FIGURE LEGEND

Supplementary Fig. S1. Alignment of the amino acid sequences of HpuA from carriage and disease isolates of *Neisseria meningitidis*. Full-length sequences were generated from two disease isolates (z2491 and 8047) and six carriage isolates (N222, N117, N119, N134, N114, N88 and N138). The sequences were translated from the first codon after the repeat tract and so do not include the initiation codon, signal sequence and repeat tracts. These sequences were then aligned with HpuA sequences derived from the published genome sequences of strains Z2491 (Parkhill *et al.*, 2000) and FAM18 (Bentley *et al.*, 2007).

Supplementary references

Bentley, S. D., Vernikos, G. S., Snyder, L. A. S. and other authors (2007). Meningococcal genetic variation mechanisms viewed through comparative analysis of serogroup C strain FAM18. *PLoS Genet* **3**, e23. http://dx.doi.org/10.1371/journal.pgen.0030023

Parkhill, J., Achtman, M., James, K. D. and other authors (2000). Complete DNA sequence of a serogroup A strain of *Neisseria meningitidis* Z2491. *Nature* **404**, 502–506. http://dx.doi.org/10.1038/35006655
